# Emerging roles of cytoskeletal proteins in regulating gene expression and genome organization during differentiation

**DOI:** 10.1080/19491034.2020.1742066

**Published:** 2020-03-25

**Authors:** Xin Xie, S. Raza Mahmood, Tamara Gjorgjieva, Piergiorgio Percipalle

**Affiliations:** a Science Division, Biology Program, New York University Abu Dhabi (NYUAD), Abu Dhabi, United Arab Emirates; b Department of Biology, New York University, New York, NY, USA; c Department of Molecular Biosciences, The Wenner-Gren Institute, Stockholm University, Stockholm, Sweden

**Keywords:** Cytoskeletal proteins, actin, nucleus, gene expression, transcriptional reprogramming, chromatin regulation, genome organization, development and differentiation

## Abstract

In the eukaryotic cell nucleus, cytoskeletal proteins are emerging as essential players in nuclear function. In particular, actin regulates chromatin as part of ATP-dependent chromatin remodeling complexes, it modulates transcription and it is incorporated into nascent ribonucleoprotein complexes, accompanying them from the site of transcription to polyribosomes. The nuclear actin pool is undistinguishable from the cytoplasmic one in terms of its ability to undergo polymerization and it has also been implicated in the dynamics of chromatin, regulating heterochromatin segregation at the nuclear lamina and maintaining heterochromatin levels in the nuclear interiors. One of the next frontiers is, therefore, to determine a possible involvement of nuclear actin in the functional architecture of the cell nucleus by regulating the hierarchical organization of chromatin and, thus, genome organization. Here, we discuss the repertoire of these potential actin functions and how they are likely to play a role in the context of cellular differentiation.

## Introduction

In eukaryotic cells, the cytoskeleton comprises a network of dynamic filaments which extends form the cytoplasm to the nucleus and supports diverse cellular functions in many cellular compartments. Among the cytoskeletal proteins, actin is probably the most abundant and highly conserved protein and its role in regulating cell shape, motility and cytoskeletal organization has been extensively studied [1]. Cytoplasmic actin forms the basis of the cytoskeleton and regulates cellular processes, such as cell motility and adhesion via controlled polymerization of monomers (G-actin) into filaments (F-actin) [2]. Actin also plays critical roles inside the nucleus; there is evidence that the levels of nuclear actin are actively regulated [3] and disruption of proteins involved in nuclear actin transport can lead to embryonic lethality [2]. Similar to the cytoplasmic actin pool, nuclear actin appears to retain its polymerization properties. More than 30 actin-binding proteins that affect actin polymerization are present inside nuclei [4] and since dynamic nuclear actin polymerization has been observed [5], the role of nuclear actin binding proteins in regulating the polymerization state of nuclear actin is an area of active research.

While it has long been known that actin is present in the nucleus of various cell types [6–8], discriminating nuclear and cytoplasmic actin has remained challenging. Biochemical and imaging studies have now provided convincing evidence that nuclear actin plays crucial roles in nuclear processes such as transcriptional regulation [9,10], chromatin regulation [11] and long-range chromatin movements [12]. There is evidence suggesting that actin regulates transcription by directly affecting the activity of RNA polymerases (RNAPs) with roles in transcription initiation, elongation and ribosome biogenesis [2,9,13–15]. Further, there is evidence that polymeric actin may act in concert with nuclear myosin I to drive RNA polymerase I transcription [15]. However, whether the monomeric or polymeric form of actin is involved in regulating other RNA polymerases remains to be investigated. It has also been shown that actin preferentially associates with euchromatic regions and interacts with ribonucleoprotein complexes that control splicing and transport of nascent transcripts [16]. Consistent with its role in transcriptional regulation, actin seems to mediate long range chromatin movements and perturbation of actin polymerization inhibits movement of fluorescently labeled transgenes, endogenous chromatin territories and chromosome segregation [2,12]. Furthermore, actin affects chromatin organization by associating with or being a component of developmentally important chromatin remodelers such as INO80 and TIP60 complexes in yeast and BAF (Brahma-associated factors) complex in mammals [1]. As the BAF complex has important roles in lineage specification and cell fate reprogramming, actin depletion has also been shown to affect the induction of differentiation programs in cells undergoing transcriptional reprogramming [17]. Emerging evidence also shows that mechanical force can affect actin dynamics and chromatin regulation [18]. Finally, recent studies have also reported considerable cross-talk between actin and microtubule cytoskeletons in regulating critical processes such as cell migration, cell division, cell polarity and neuronal shape and function [19].

Here, we focus on the involvement of cytoskeletal proteins in regulating differentiation and development. We will first review evidence for the roles of cytoskeletal proteins in signaling and transduction and then focus on their nuclear functions. We will then describe various approaches that have been utilized for targeting cytoskeletal proteins inside the nucleus and discuss neurogenesis as a paradigm to study nuclear actin’s function during cell differentiation. Finally, we will outline future research directions with particular emphasis on the potential role of nuclear actin in regulating 3D genome organization.

## The cytoskeleton and signal transduction

**Figure 1.**
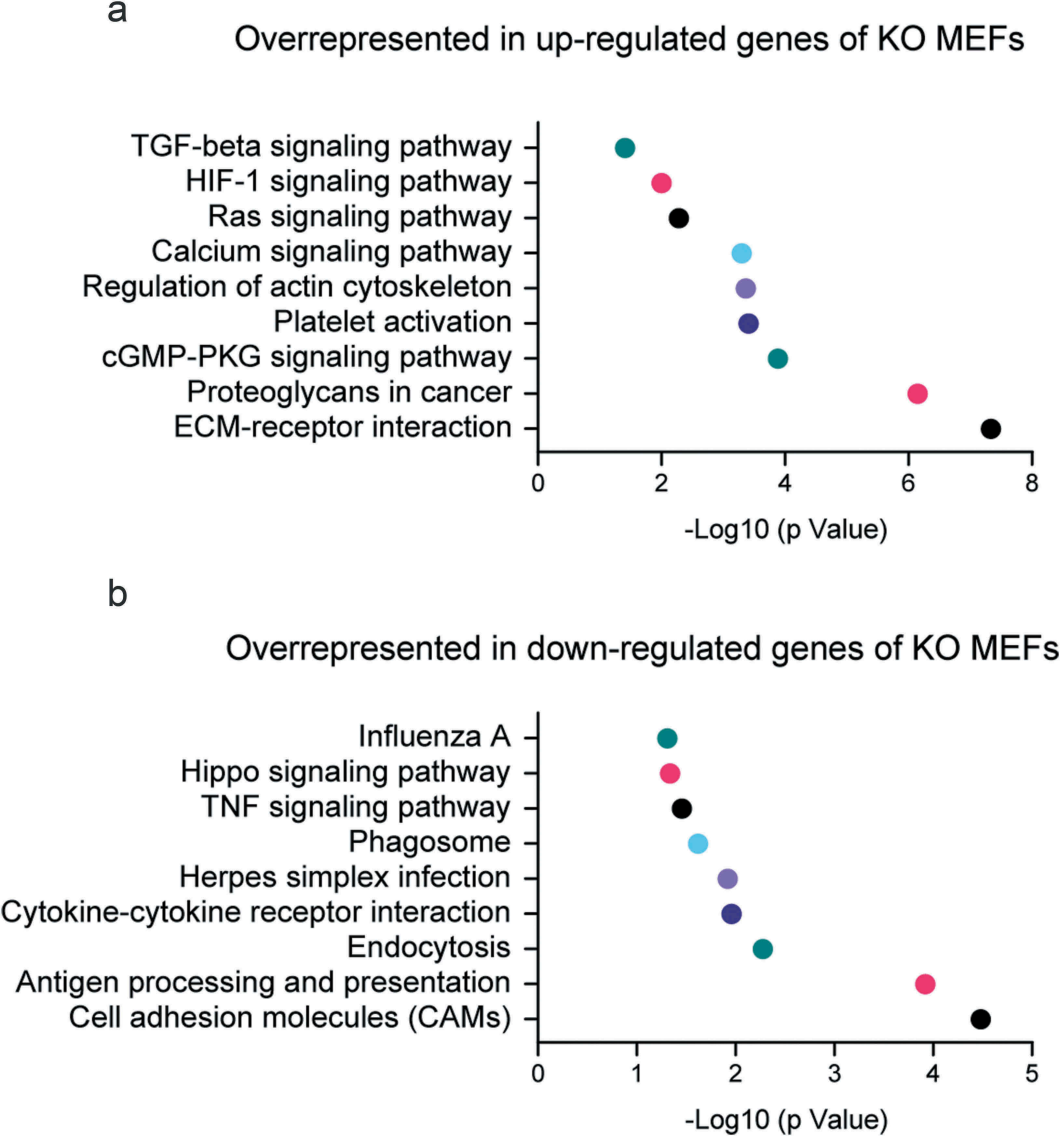
KEGG pathways significantly enriched in the differentially expressed genes between beta-actin WT and KO MEFs. Genes significantly up-regulated or down-regulated (Padj<0.05, fold change >2) in KO cells were subject to enrichment analysis using DAVID Bioinformatics Resources. Selected KEGG pathways overrepresented in up-regulated genes or down-regulated genes are displayed in **(A)** and **(B)** respectively.

The cytoskeleton needs to be spatially reorganized during cell differentiation, which is critical to maintain morphology and fulfill specific cellular functions. Cytoskeleton rearrangements and dynamics are often accompanied by changes in certain signal transduction pathways during cell differentiation and morphogenesis [20–23]. A classic example is the regulation of nuclear translocation of MAL (MTRF-A), a Myocardin family transcription factor [24], by dynamic changes of the actin pool. In the nucleus, MAL functions as a cofactor of transcription factor SRF (Serum Response Factor), and the activation of their transcription activity is a key event in cell differentiation processes [25]. It has been demonstrated that monomeric G-actin inhibits nuclear translocation of MAL while increased F-actin polymerization favors its nuclear import [26,27]. Forced overexpression of nucleus-localized, non-polymerizable form of the actin mutant R62D^NLS^ can decrease the mobility of neuron cells by regulating SRF transcription activity [28]. Moreover, the clustering of signaling proteins by F-actin mediated-microdomain formation in dendritic spines and during T cell signaling are some of the many possible examples of microfilament regulation of signaling pathways [29–31]. We also demonstrated that the cytoskeleton reorganization in β-actin knockout mouse embryonic fibroblasts (MEFs) causes changes in the biophysical properties of the plasma membrane and the activation of TGFβ signaling pathway (Figure 1) [32,33]. Recent studies start to reveal that multiple signaling proteins tend to form high-order machinery for spatial control of efficient signal transduction [34,35]. Since proteins or protein complexes associated with actin fibers are subject to mechanical stretch or spatial relocation due to cytoskeleton rearrangement, future efforts are needed to elucidate how the signaling machinery and the signal transduction processes are regulated by actin filament reorganization during cell differentiation.

Another interesting perspective is that the actin cytoskeleton is linked with the nuclear lamina via the LINC complex, which serves as a sensing machinery to mechanical signals [36,37]. Since the nuclear lamina plays a central role in chromatin organization by forming the anchor of Lamina-Associated Domains (LADs) [38], dynamic changes of the actin cytoskeleton upon mechanical stress can potentially affect chromatin organization. Indeed, in human epidermal keratinocytes mechanical forces cause defective heterochromatin anchoring to the nuclear lamina by inducing actin polymerization [39]. This seems to be mediated by the enrichment of NMIIA (Non-muscle myosin II A) and actin-capping protein emerin at the nuclear periphery, which ultimately results in global chromatin redistribution [39]. Interestingly, lamin A/C has been recently shown to employ perinuclear actin cables to maintain nuclear morphology under mechanical stress [40]. Moreover, emerin together with nuclear lamins, is required for the spatial organization of chromosome territories in response to mechanical cues [41]. Depletion of lamin A/C and emerin causes actin fiber formation, increased levels of nuclear myosin 1 (NM1) and chromatin reorganization [42]. Therefore, the mechanosensory pathway via actin and protein components of the nuclear lamina can regulate chromatin organization, gene expression and cell lineage commitment in response to extracellular mechanical stimuli [39].

Microtubules are also linked with multiple signaling pathways, directly affecting nuclear functions. One of the best characterized examples of microtubules involvement in signal transduction is Costal 2 in *Drosophila* development. Costal 2 was first identified as a suppressor of the hedgehog signaling pathway [43]. Subsequently, sequence alignment revealed its similarity to kinesin heavy chains [44]. Biochemical analysis demonstrated the formation of a large complex consisting of Costal 2 and other hedgehog pathway components, including the protein kinase Fused and the transcription factor Cubitus Interruptus (CI). In the absence of hedgehog signal, this complex seems to be sequestered in the cytoplasm by interacting with microtubules via Costal 2. When treated with hedgehog, the complex shows reduced affinity for microtubules, releasing the transcription factor CI for its nuclear function [44]. Furthermore, multiple kinases were reported to interact with the microtubule network. For example, MLK2 (Mixed-lineage kinase 2), a MAPK kinase kinase-like protein involved in activation of the JNK pathway, was found to interact with members of the kinesin-like KIF3 family [45]. The extracellular signal regulated kinase ERK1 and ERK2 were also reported to interact with microtubules [46,47]. Wnt signaling pathway kinase GSK-3β (Glycogen synthase kinase-3β) physically associates with microtubules and can phosphorylate several microtubule-associated proteins (MAPs) [48–50]. However, the biological significance of these microtubule-kinase interactions remains unclear. It is therefore important to further investigate how microtubules dynamics affect signal transduction during cell differentiation and potentially address whether coordination of microtubules and the actin cytoskeleton is important not only in several types of cell movement but also during development and differentiation.

## Cytoskeletal proteins in development and cell differentiation: a nuclear perspective

Based on the above considerations, it is not surprising that pharmaceutical or genetic perturbation of microfilament and microtubule dynamics can affect differentiation of cells that require dramatic morphological changes, such as in the case of myogenesis and neurogenesis [51–54]. Manipulation of actin dynamics also affects osteogenic or adipogenic differentiation in mesenchymal stem cells [22,55], and modulates mesodermal and endodermal lineage differentiation in pluripotent stem cells [56]. Since cytoskeletal alterations affect the nuclear level of actin or tubulin, it is essential to investigate the involvement of cytoskeletal proteins in cell differentiation and development from a nuclear perspective.

Both actin and tubulin have been found to shuttle between cytoplasm and nucleus [57–59]. An emerging concept of ‘nucleoskeleton’ has been proposed to play a role in genome organization and gene regulation in cell differentiation and development [60]. Intranuclear actin has been identified in the oocytes of fruit fly, avian and amphibian species, and the early mouse embryo [61–63], suggesting an evolutionarily conserved function of nuclear actin in early embryogenesis. In *Drosophila* oogenesis the formation of nuclear actin rod in germinal vesicle and nurse cells is regulated by Fascin [63], although its biological function remains unclear. In *Xenopus* oocytes, the presence of large amount of nuclear actin seems to be required for stabilizing nuclear architecture and transcription [62,64]. The actin-nucleation protein WAVE1, which is also present in the nuclei of *Xenopus* oocytes, is essential for early embryogenesis since the expression of *Hox* genes is downregulated when WAVE1 is knocked down [65]. The nuclear function of WAVE1 is further supported by the rescue of *Hox* genes expression when WAVE1 is reintroduced into the nucleus. In mammalian cells, actin-polymerization by nuclear N-WASP is also required for the induction of *HoxB* by retinoic acid [66]. A critical question is: why does actin need to polymerize in the nucleus to facilitate the transcription of specific genes during early embryogenesis? This seems to be relevant to specific developmental processes since nuclear actin rods are only present at certain developmental stages. The polymerized nuclear actin is very dynamic and different from the cytoskeletal actin fibers [67]. As polymerized actin, rods observed in the nuclei of cellular models of disease pathologies can alter the distribution of chromatin and RNA polymerase II [68], a speculation is that polymeric actin may be involved in the establishment of a favorable chromatin state compatible with gene expression during embryogenesis.

**Figure 2.**
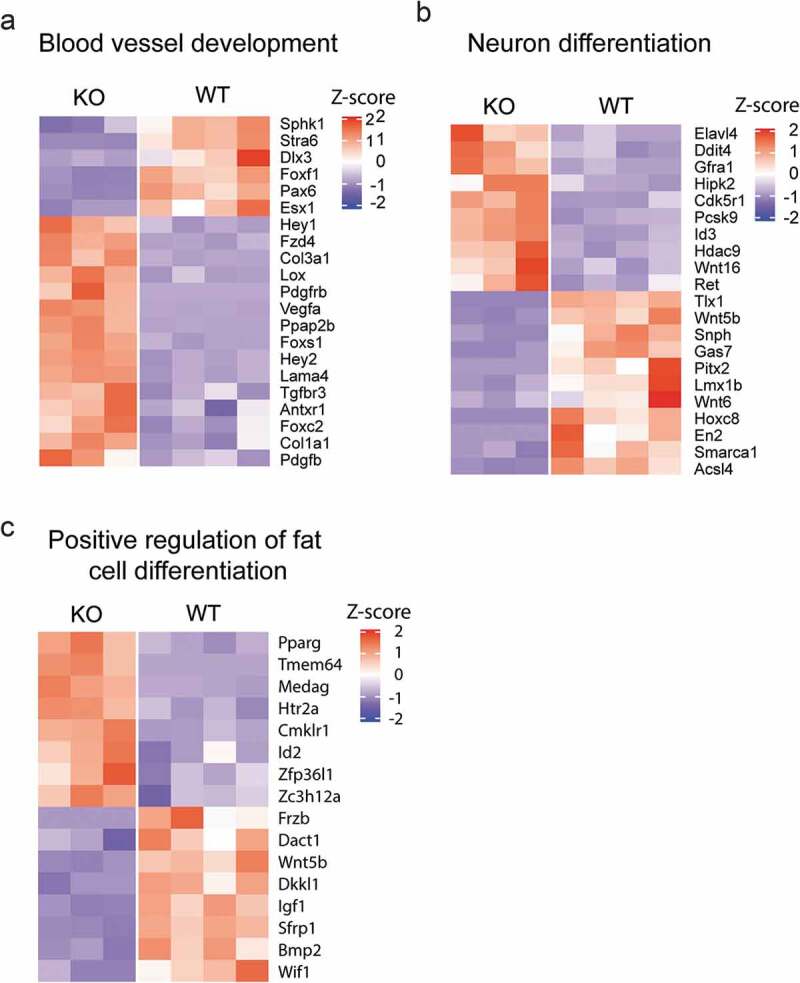
Cell fate-related genes are differentially expressed between beta-actin WT and KO MEFs. Genes differentially expressed (Padj<0.05, fold change >2) were subject to Gene Ontology (GO) enrichment analysis using DAVID Bioinformatics Resources. The expression level of genes associated with (a) Blood vessel development, (b) Neuron differentiation and (c) Positive regulation of fat cell differentiation are shown.

Accumulating evidence also demonstrates the important role of nuclear actin in differentiation of several cell types. Cytochalasin D treatment causes rapid accumulation of nuclear actin and enhances osteogenic differentiation of mesenchymal stem cells (MSC) [69]. Intranuclear actin induces the nuclear export of Yes-associated protein (YAP), releasing the osteogenic transcription factor RUNX2 from the inhibition by YAP [69]. Through pharmaceutical or genetic perturbation of actin dynamics, it was further demonstrated that increased nuclear actin levels not only affect nuclear morphology, but also that the intranuclear actin structure seems to regulate the lineage decision in MSC. Actin filament branching by the Arp2/3 complex is required for osteogenesis while inhibiting actin branching stimulates adipogenesis [70]. During the differentiation of HL-60 cells into macrophages, the nuclear translocation of actin is observed and is involved in the transcriptional activation of macrophage-related genes [71]. In epidermal progenitor cells (EPCs), the mechanical force induced by actin polymerization causes the reduction of nuclear actin level, which regulates the lineage commitment [39]. Interestingly, the reduced nuclear actin level leads to increased H3K27me3 level and impaired RNA Pol II activity, as well as global chromatin reorganization. We recently showed that in β-actin wild type, heterozygous and knockout MEFs, different dosages of actin present in the nucleus can differentially influence histone marks such as H3K9Me3 and H3K4Me3, which regulate the expression of specific gene programs and cell identity [72]. For examples, the loss of β-actin causes the dysregulation of genes involved in blood vessel development, neuron differentiation and fat cell differentiation in MEFs (Figure 2). Therefore, dynamic changes of intranuclear actin possibly regulate chromatin organization, epigenetic modifications and gene transcription.

Although there is compelling evidence for the involvement of nuclear actin in the differentiation of multiple cell types, several key questions remain unclear. Addressing dynamic changes in intranuclear actin levels or structure during differentiation and development ideally requires the use of animal models to gain *in vivo* insights. Second, it is important to understand how nuclear actin levels regulate chromatin organization. One possible mechanism is that nuclear actin regulates the activity of chromatin-remodeling complexes since actin is a component of several well-characterized chromatin remodeling complexes [73]. Clarifying exactly which complexes are regulated by nuclear actin levels can provide valuable insight. Finally, future efforts should be made to develop better experimental systems that differentiate the cytoplasmic function from the nuclear function of cytoskeletal proteins.

## Manipulating the nuclear pool of cytoskeletal proteins in genome organization during development

Manipulating the nuclear pool of cytoskeletal proteins without perturbing cytoplasmic actin function is not an easy task, as cytoskeletal proteins such as actin are the most abundant proteins in the cell [74]. Nevertheless, there are several approaches for targeting cytoskeletal proteins in the nucleus, including (i) microinjection of antibodies, (ii) genetic perturbation of actin import or export factors and (iii) tagging actin with a nuclear localization signal (NLS).

Early efforts in manipulating nuclear actin were performed with somatic nuclear transfer model to study the role of nuclear actin in transcriptional reprogramming [65,75]. The somatic nucleus transferred into germinal vesicle of *Xenopus* oocyte can undergo reprogramming within 2 days, a process accompanied by nuclear actin polymerization [75]. By microinjecting a monoclonal anti-actin antibody into the germinal vesicle to inhibit nuclear actin polymerization, researchers demonstrated the essential role of nuclear actin polymerization in the induction of the *Oct4* gene during the nuclear reprogramming [75]. Since the discovery of the importin-9 and exportin-6 as the nuclear import and export factors respectively [3,57], several groups managed to modulate nuclear actin levels by perturbing nuclear actin import or export. In mesenchymal stem cells (MSC) silencing importin-9 inhibits adipogenesis, indicating the maintenance of nuclear actin level is necessary for adipogenic lineage [70]. In epidermal progenitor cells (EPCs) the mechanical force induces the reduction of nuclear actin level, which is accompanied by H3K27Me3 increase and RNA Pol II activity inhibition [39]. RNAi-mediated silencing of exportin-6 can rescue both the H3K27Me3 change and RNA Pol II activity by maintaining a high level of nuclear actin [39]. Actin tagged with NLS has been shown to play a role in the activation of genes rather than gene repression in Hela cells [76]. However, over-expression of β-actin with NLS in HaCaT keratinocytes leads to the down-regulation of adhesive and cytoskeletal genes [77].

Studying the functions of cytoskeletal proteins during development is hindered by the fact that the knockout animals can be embryonic lethal, as is the case with β-actin knockout mice [78]. Recently established reprogramming protocols using small chemicals provide alternative models to investigate how the lack of β-actin may affect cell differentiation from a nuclear perspective. Direct reprogramming of somatic cells into functional neurons or neural stem cells have been well-established in mouse and human cells [79–82]. Mouse skeletal myofibroblast and human dermal fibroblasts were successfully reprogrammed into adipocytes by different chemicals [83,84]. Chemical reprogramming can also convert somatic cells into pluripotent stem cells [85]. These established direct reprogramming methods therefore allow us to study the potential role of nuclear β-actin in controlling the state of chromatin and, consequently, expression of gene programs during cell fate changes using fibroblasts from β-actin knockout embryos as models.

## Neurogenesis as a paradigm to study the role of actin in genome organization during development and differentiation

Actin-containing structures play a role in neuronal development, including growth cone dynamics, remodeling of dendritic spines and migration of neuronal precursors [86–88]. While β-actin and γ-actin are both expressed in mammalian neurons, they exhibit different localization patterns and dynamics during neuronal development: γ-actin is expressed evenly in the cell body, dendrites and axons, while β-actin is localized to structures with high capacity for remodeling like the growth cone [89,90]. β-actin is also important in overall nervous system development. Ablation of β-actin in the central nervous system (CNS) in mice results in histological abnormalities in the hippocampus and cerebellum and leads to localized defects in axonal crossing of the corpus callosum [91], while β-actin-null neural crest cells exhibit elevated apoptosis and impaired migration [92].

**Figure 3.**
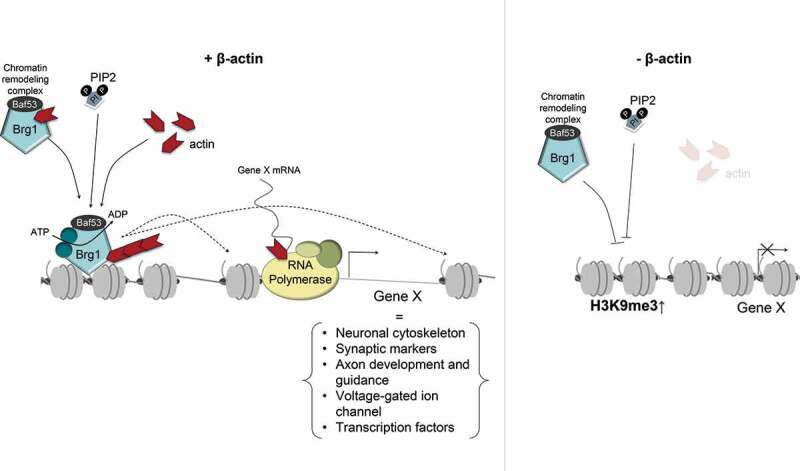
Speculative model describing the involvement of nuclear β-actin in epigenetic regulation during neuronal reprogramming. (a) In wild type cells, β-actin is a component of the BAF chromatin remodeling complex and it is required for the activity of its ATPase subunit Brg1 and chromatin association around the transcription start sites. Phosphoinositide-4,5-bisphosphate (PIP2) binding may cause the association of BAF complex with nuclear actin filament and chromatin. The activated BAF complex, mobilized by the dynamic assembly and disassembly of the nuclear actin filament, is recruited to the chromatin where it modifies nucleosomes in an ATP-dependent manner. BAF-dependent chromatin re-modeling is required to maintain a favorable chromatin status for the induction of neuronal genes during reprogramming. (b) Loss of β-actin, impairs activity and recruitment of Brg1 and a functional BAF complex, resulting in impaired chromatin remodeling and increased chromatin compaction at the transcription start sites of many neuron-related genes with increased H3K9Me3 and H3K27Me3 levels. This increase in heterochromatin forms an epigenetic barrier for their expression during transcriptional reprogramming.

Recent work has shown that the nuclear β-actin pool significantly contributes to neuronal development by regulating genome organization and activation of neuronal programs during neurogenesis. To address this question, we directly reprogrammed wild-type and β-actin knockout embryonic fibroblasts as well as embryonic fibroblasts from a heterozygous mouse where only one of the β-actin alleles was disrupted [17]. Compromising β-actin levels was associated with impaired expression of neural and proneural genes involved in the neuronal cytoskeleton, synapse, axon development and guidance, voltage-gated ion channels and neuron-related transcription factors. Notably, members of the *Zic* and *Irx* family of genes, which are involved in the onset of neuronal fate [93], were severely down-regulated in the absence of β-actin [17]. The overall impaired induction of neuronal genes in β-actin knockouts was correlated with increased H3K9Me3 levels and loss of Brg1 (ATPase subunit of BAF chromatin remodeling complex) occupancy at transcription start sites of multiple gene loci, including *Zic* and *Irx* genes. Results from this study suggest that β-actin regulates a heterochromatin landscape required for the optimal induction of neuronal gene programs during direct reprogramming. In the absence of nuclear β-actin, the chromatin association of Brg1 is abrogated, suggesting a loss of BAF localization and function at the regulatory region of neuronal genes. A working model is that the defective chromatin remodeling and the elevated H3K9Me3 level at transcription start sites hinder the neuronal gene expression during neurogenesis due to loss of chromatin accessibility for the transcription machinery (Figure 3). These finding is consistent with the rising notion that H3K9Me3-based heterochromatin creates an epigenetic barrier during cell fate change [94]. We, therefore, propose a model whereby nuclear β-actin primarily contributes to neuronal differentiation by presetting chromatin to ensure expression of developmental genes, including pioneer transcription factors. This working model is in line with a role for nuclear actin in the maintenance of heterochromatin levels, possibly by counteracting the role of polycomb repressive complexes [60,95]. Future work in other differentiation models will possibly establish if this is a general mechanism.

## Nuclear actin and the 3D genome: a future research direction

Genome architecture is the result of multiple layers of chromatin folding. The chromosomes of eukaryotes are folded into a multi-layered hierarchical structure with each layer playing a role in regulating genome activity and function. Recent studies have utilized chromosome conformation capture techniques like 3-C and Hi-C to infer 3D genome organization by analyzing interaction frequencies between different regions of the genome [96]. Such studies have revealed a genome organization in which chromosomes occupy distinct territories in nuclear space and then fold into gene-rich and transcriptionally active or gene-poor & transcriptionally repressed compartments (referred to as A and B compartment respectively) [97]. These compartments are further divided into topologically associated domains (TADs) that are enriched for local genomic interactions and conserved across cell types and species. TADs further give rise to sub-TADs and chromatin loops which often vary in a tissue specific manner [96].

While there is currently no direct evidence implicating actin in compartment or TAD level 3D genome organization, several studies have confirmed the role of nuclear actin in regulating chromatin structure at a local level and hinted at a potential role in 3D genome organization. It has been shown, for instance, that inhibition of actin polymerization can increase the volume and surface area of chromosomal territories [98]. Similarly, recent work from our lab shows that β-actin deficient cells exhibit changes in the spatial organization of H3K9Me3/HP1α-positive heterochromatin [72]. The observation that cells lacking β-actin show both changes in spatial organization of heterochromatin and dysregulation of specific gene expression programs during transcriptional reprogramming suggests that actin deficiency may induce defects in 3D genome architecture. Although any direct role for actin in regulating higher order chromatin structure is yet to be elucidated, it is possible that potential changes in 3D genome organization may result from changes in activity of chromatin remodeling complexes dependent on actin function.

Two such molecules potentially affected by actin are HP1α and Polycomb Repressive Complex 1 (PRC1). HP1α and PRC1 are characteristic of constitutive and facultative heterochromatin respectively and both have been shown to form liquid-like droplets in vivo and in vitro [99–103]. Since the 3D arrangement of chromatin fibers and separation of genomic regions into compartments is thought to arise from a phase separation-based mechanism that allows self-organization of chromatin domains [104], the ability of HP1α and polycomb proteins to form phase separated bodies makes them ideal candidates for a role in regulating higher order chromatin structures. Furthermore, HP1α protein is known to homodimerize and bridge H3K9 methylated nucleosomes while PRC1 proteins are thought to induce clustering of polycomb bound chromatin and nucleosomes into phase separated ‘polycomb bodies’ implicated in the regulation of genome architecture [105] and maintenance of long-range chromatin interactions [106]. β-actin depletion can potentially affect the regulation of both HP1α and PRC1 proteins [17,72]. We have shown that β-actin knockout cells exhibit redistribution of HP1α-positive heterochromatin and loss of chromatin association of Brg1, the essential ATPase subunit of the BAF complex [72]. Since Brg1 directly opposes the deposition of polycomb proteins [95], these observations support a model where loss of β-actin may trigger increased recruitment of PRC complexes to specific genomic loci and potentially induce compartment level changes in heterochromatin organization.

In addition to potential changes in compartment level organization, loss of Brg1 chromatin binding in β-actin KO cells may also affect genome architecture at the level of TADs and loops [107–109]. It has been shown that more than 20% of Brg1 binding sites overlap with TAD boundaries and Brg1 loss at these boundaries can lead to a decrease in TAD insulation [107]. Similarly, it has also been shown that Brg1 binding sites overlap with distal enhancers and loss of Brg1 binding can impair enhancer activation and cell differentiation [108]. Collectively, the alterations of Brg1 and PRC activity in the absence of nuclear actin are intriguing possibilities by which actin may affect genome organization. Such a role is yet to be elucidated and is an important area for future research.

## Concluding remarks

Cytoskeletal proteins are emerging as key regulators of nuclear functions and they impact key cellular processes such as differentiation and development. Among these cytoskeletal proteins, the nuclear functions of actin and a few forms of myosin are probably the best characterized. Using both loss-of-function and gain-of-function model systems for nuclear actin, we now appreciate a fundamental role for nuclear actin in regulating heterochromatin, both in terms of segregation at the nuclear lamina and maintenance of heterochromatin levels in the nuclear interior. These observations are compatible with a role for nuclear actin in consolidating genome architecture while promoting expression and/or repression of gene programs that are fundamental for cellular function. These mechanisms are particularly important in development and differentiation. In fact, a knockout mouse model for β-actin is embryonic lethal at stage E10.5 immediately before key developmental pathways such as neurogenesis are initiated. While recent evidence hints at a role for nuclear actin in 3D genome organization, whether actin consolidates genome architecture and contributes to the hierarchical organization of chromatin is not known. Experiments utilizing conformation capture techniques therefore offer an exciting avenue for future research. In combination with direct transcriptional reprograming techniques in actin knockout cells undergoing differentiation can also shed light on nuclear actin’s role in regulating cell fate. Similarly, the development of better experimental systems for precise manipulation of nuclear actin levels and replication of these studies in animal models are also critical for elucidating actin’s role in development and differentiation. An exciting frontier ahead of us is also to find out the impact of these mechanisms in the 3D development of tissues and the emerging use of organoids may be a suitable model to address these questions in combination with genome-wide approaches.

In development and differentiation, nuclear actin engages in multiple nuclear functions while maintaining a degree of specificity. Regulated nuclear actin polymerization and depolymerization which have now been observed in the nucleus may contribute to selective interactions with specialized nuclear machinery, similarly to the cytoplasmic actin pool. For instance, nuclear actin polymerization has been suggested to evict repressive chromatin regulators while mediating recruitment of the BAF complex to upstream regulatory regions of occluded genes during oogenesis. Going forward, it will therefore be important to identify the nuclear actin interactome as a proxy to dissect the specificity of the many nuclear actin functions, including differentiation. Seeing that several loss-of-function and gain-of-function models are now available to study nuclear actin, integrating metabolomics studies with the available genome wide analyses such as transcriptional profiling will be a way forward to start identifying the implications of dysregulated nuclear actin function in physiology.
